# Supplementary material for: PBRM1 bromodomains associate with RNA to facilitate chromatin association
Source: Nucleic Acids Res. 2023 Feb 20;51(8):3631–49. doi: 10.1093/nar/gkad072 (PMC10164552; doi:10.1093/nar/gkad072)
Supplement: gkad072_Supplemental_File [file gkad072_supplemental_file.pdf]

a

```
BD1    ----------SMTV---------DPI-AVCHELYNTIRDYK-DEQGRLLCELFIRAPKRRNQPDYYEVVSQPI
BD2    ----------SMSP--------AY-LKEILEQLLEAIVVAT-NPSGRLISELFQKLPSKVQYPDYYAIIKEPI
BD3    ----------SM-------------------QLYDTVRSCR-NNQGQLIAEPFYHLPSKKKYPDYYQQIKMPI
BD4    MGDSMISSATSDTGSAKRKSK-KNIRKQRMKILFNVVLEAREPGSGRRLCDLFMVKPSKKDYPDYYKIILEPM
BD5    ----------SMSGISPKKSKYMTPMQQKLNEVYEAVKNYT-DKRGRRLSAIFLRLPSRSELPDYYLTIKKPM
BD6    ----------SMNV--------TLLIQELIHNLFVSVMSHQ-DDEGRCYSDSLAEIPAVD--PNF--PNKPPL
                 *                     :   :        *:  .  :   *     *::      *:

BD1    DLMKIQQKLKMEEYDDVNLLTADFQLLFNNAKSYYKPDSPEYKAACKLWDLYLRTRNEFVQKG----------
BD2    DLKTIAQRIQNGSYKSIHAMAKDIDLLAKNAKTYNEPGSQVFKDANSIKKIFYMKKAEIEHH-----------
BD3    SLQQIRTKLKNQEYETLDHLECDLNLMFENAKRYNVPNSAIYKRVLKLQQVMQAKKKELARRDD---I-----
BD4    DLKIIEHNIRNDKYAGEEGMIEDMKLMFRNARHYNEEGSQVYNDAHILEKLLKEKRKELGPLPDDDDMASPA-
BD5    DMEKIRSHMMANKYQDIDSMVEDFVMMFNNACTYNEPESLIYKDALVLHKVLLETRRDLEG------------
BD6    TFDIIRKNVENNRYRRLDLFQEHMFEVLERARRMNRTDSEIYEDAVELQQFFIKIRDELCKNGEI--LLSPAL
        :  *  .:    *   . :  .:  : ..*       *  :: .  : ..    : ::

BD1    ----E---------------------
BD2    -------------------------E
BD3    ----E---------------------
BD4    --------------------------
BD5    ----D---------------------
BD6    SYTTKHLHNDVEKERKEKLPKEIEED
```

b

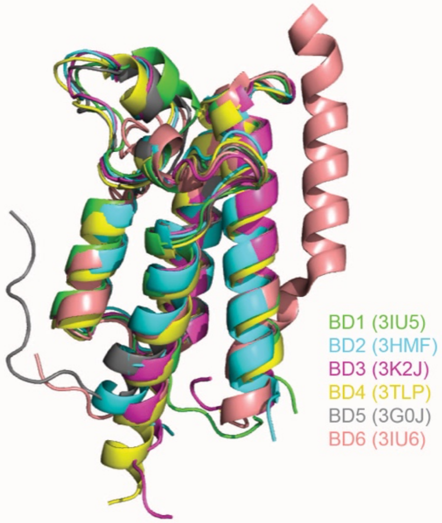


**SI Figure 1.** Sequence alignment (performed in T-Coffee) of the PBRM1 bromodomains (BD1-BD6). Included are the amino acids from the constructs used in this study. Residues are denoted as identical (* and shown in red), strongly similar (:), or weakly similar (.). Overlay of the previously solved crystal structures of BD1-BD6.

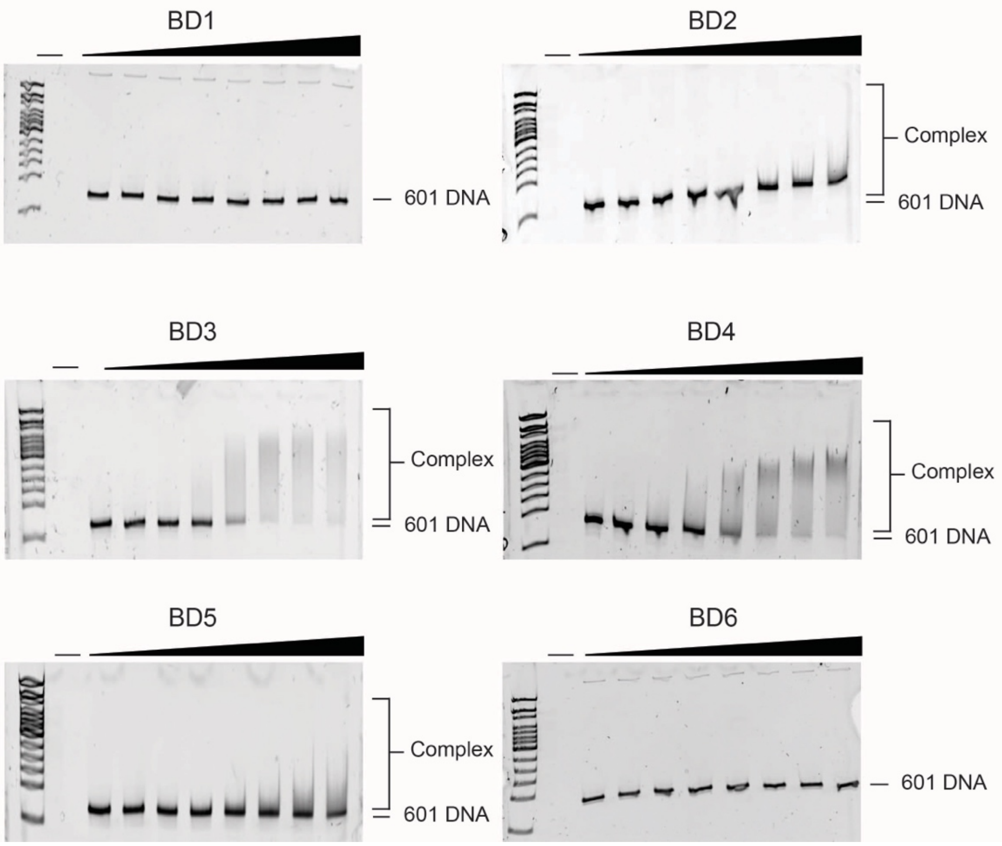


**SI Figure 2.** Electromobility shift assays (EMSAs) carried out with the Widom 601 DNA with individually purified BD1-BD6. Gels were run with a 100 bp DNA ladder on the left and stained with ethidium bromide for visualization.

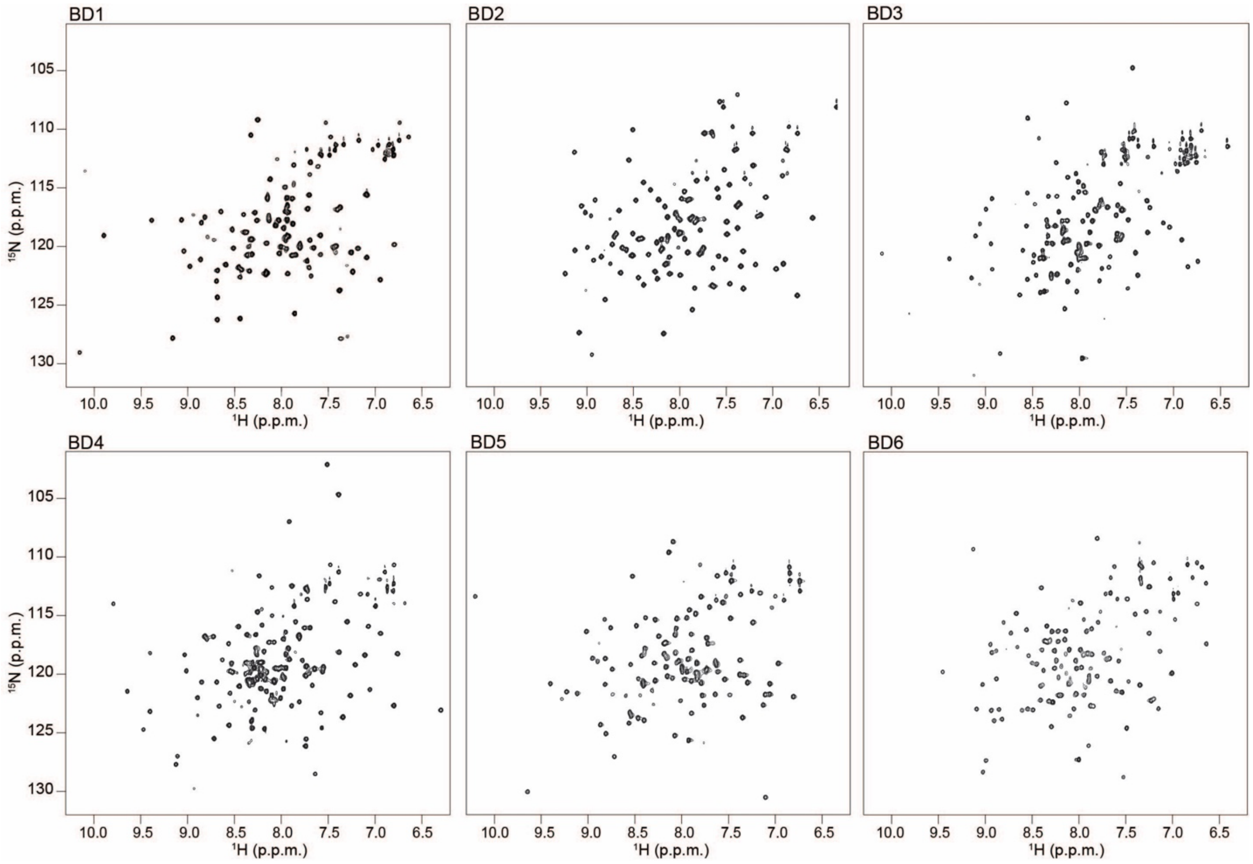


**SI Figure 3.** Individual $^{1}H$-$^{15}N$ HSQC spectra of BD1-BD6. Spectra were collected on 0.1 mM in 93% $H_2O$/7% $D_2O$ samples in 800 MHz Bruker Avance II at 25 °C.

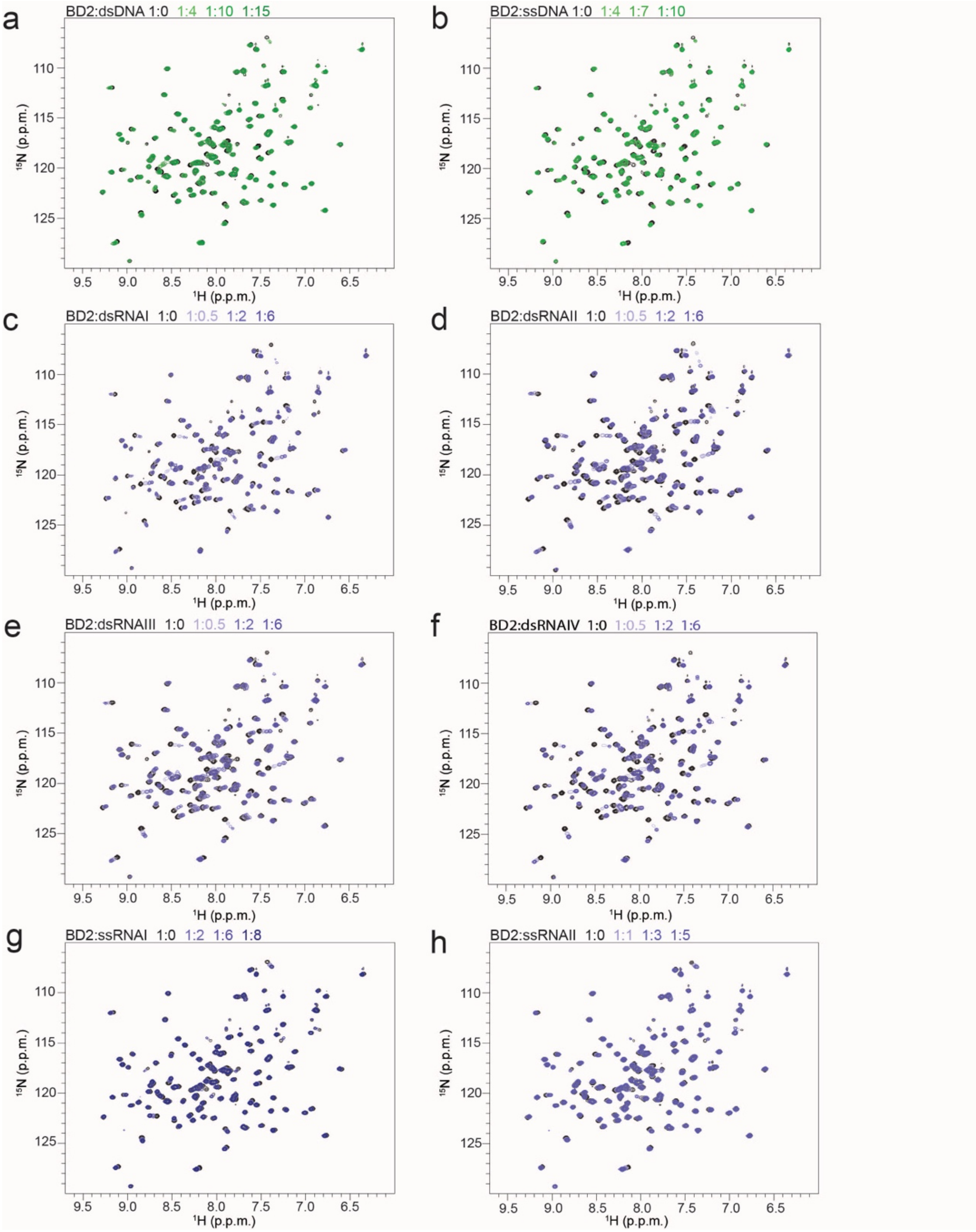


**SI Figure 4.** Overlay of $^1$H-$^{15}$N HSQC spectra of $^{15}$N-BD2 upon titration of (a) dsDNAI (b) ssDNA (c) dsRNAI (d) dsRNAII (e) dsRNAIII (f) dsRNAIV (g) ssRNAI (h) ssRNAII. Spectra are color coded according to protein:nucleic acid molar ratio as shown in the legend. dsDNA titration was collected at 1:0, 1:1, 1:2, 1:4, 1:6, 1:10, 1:12, 1:15, ssDNA was collected at 1:0, 1:0.5, 1:1, 1:2, 1:4, 1:7, 1:10, dsRNAI - dsRNAIV were collected at 1:0, 1:0.1, 1:0.25, 1:0.5, 1:1, 1:2, 1:4, 1:6, ssRNAI was collected at 1:0, 1:1, 1:2, 1:4, 1:6, 1:8 and ssRNAII was collected at 1:0, 1:0.5, 1:1, 1:2, 1:3, 1:4, 1:5. Protein concentration was 100 μM. For clarity, only 4 points are displayed.

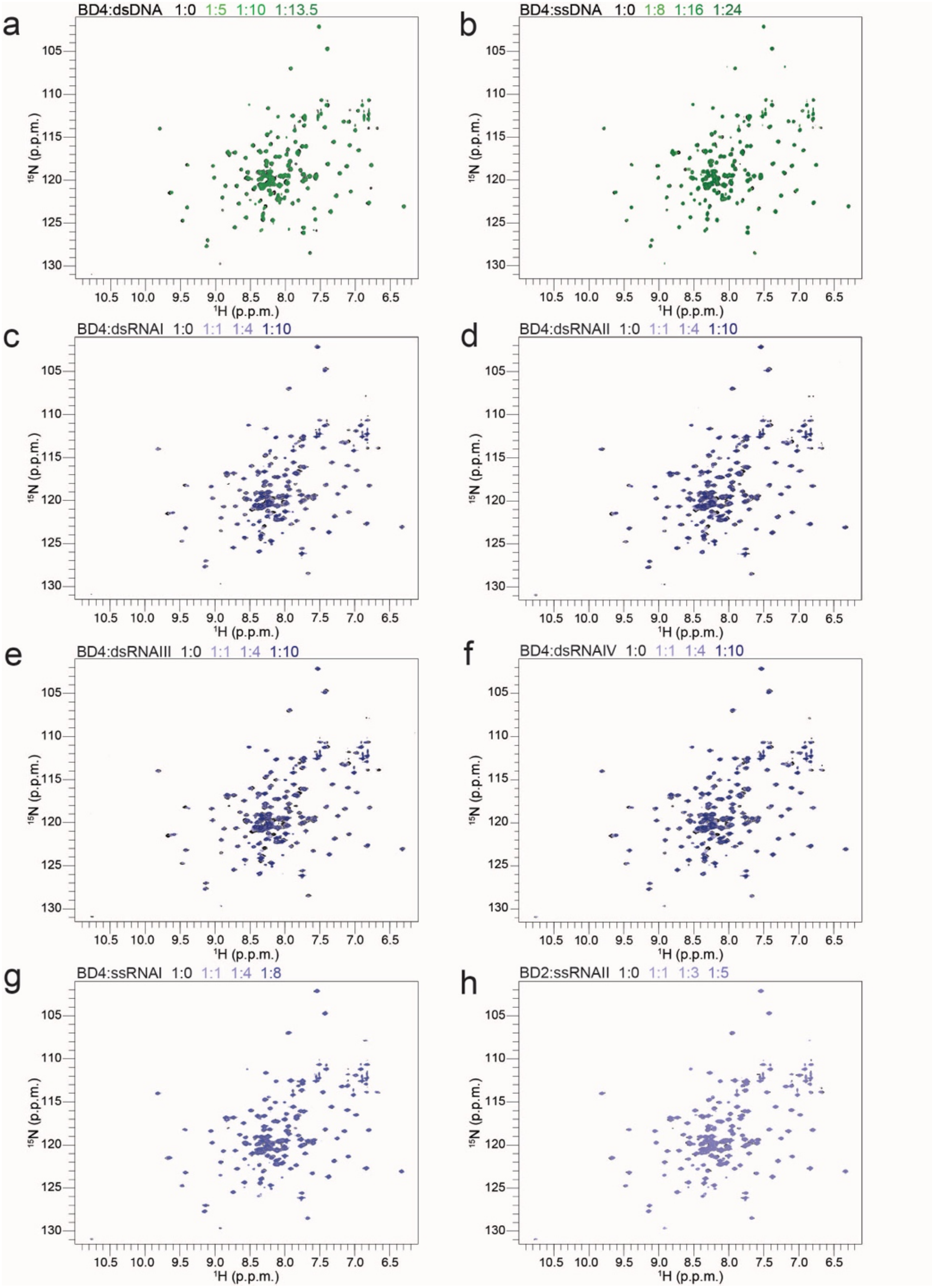


**SI Figure 5.** Overlay of $^{1}$H-$^{15}$N HSQC spectra of $^{15}$N-BD4 upon titration of (a) dsDNAI (b) ssDNA (c) dsRNAI (d) dsRNAII (e) dsRNAIII (f) dsRNAIV (g) ssRNAI (h) ssRNAII. Spectra are color coded according to protein:nucleic acid molar ratio as shown in the legend. dsDNA titration was collected at 1:0, 1:0.5, 1:1, 1:2, 1:2.5, 1:5, 1:7.5, 1:10, 1:13.5, ssDNA was collected at 1:0, 1:0.5, 1:1, 1:4, 1:8, 1:12, 1:16, 1:24, dsRNAI - dsRNAIV were collected at 1:0, 1:0.5, 1:1, 1:2, 1:3, 1:4, 1:6, 1:10, ssRNAI was collected at 1:0, 1:1, 1:2, 1:4, 1:6, 1:8 and ssRNAII was collected at 1:0, 1:0.5, 1:1, 1:2, 1:3, 1:4, 1:5. Protein concentration was 0.1 mM. For clarity, only 4 points are displayed.

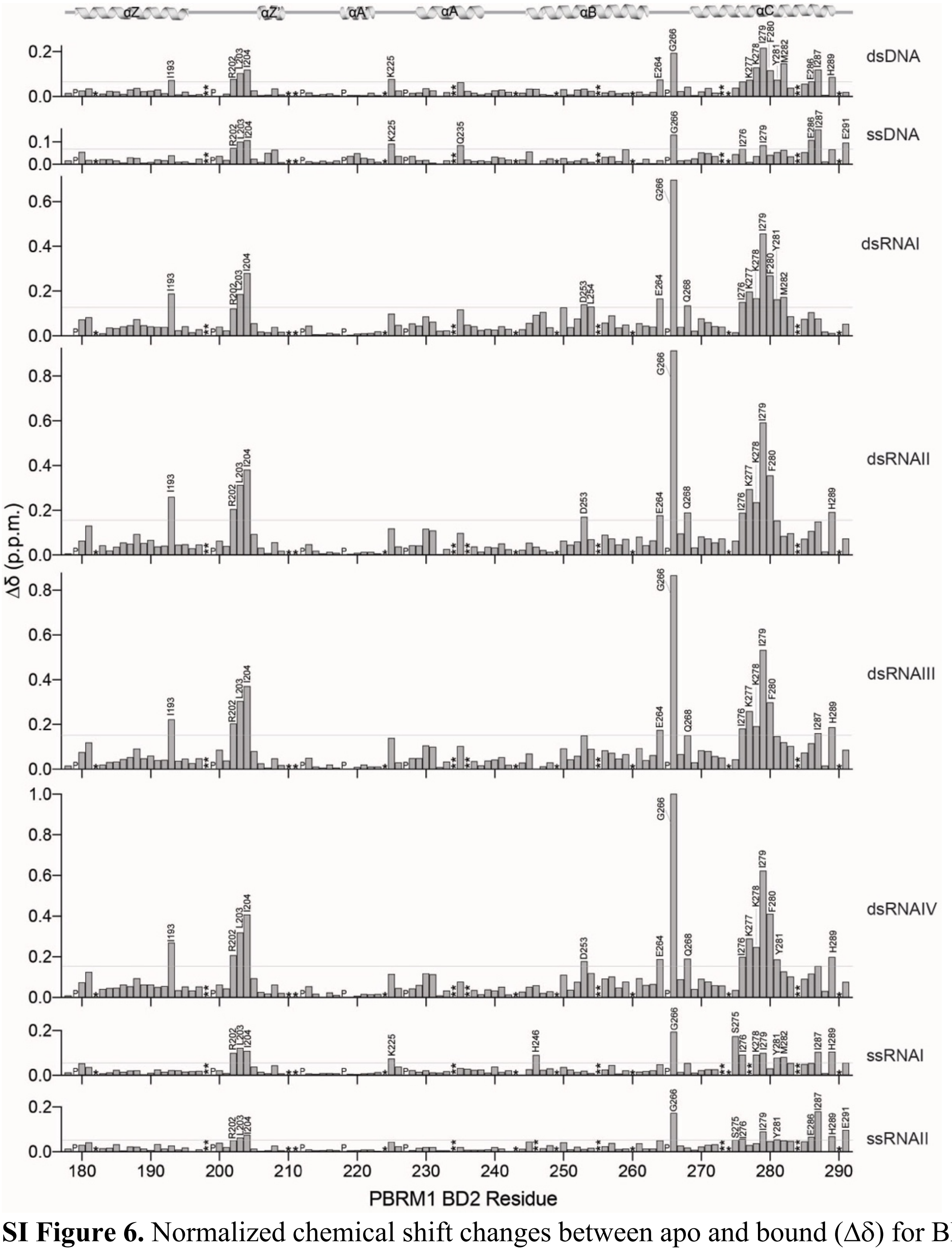


**SI Figure 6.** Normalized chemical shift changes between apo and bound (Δδ) for BD2 are plotted as a function of residue in the presence of dsDNAI, ssDNA, dsRNAI, dsRNAII, dsRNAIII, dsRNAIV, ssRNAI and ssRNAII (from top to bottom). Final molar ratios are 1:15, 1:10, 1:6, 1:6, 1:6, 1:6, 1:8 and 1:5, respectively. Residues that are unassigned or untrackable due to overlap are marked as (*) and (**) respectively. The secondary structure of the PBRM1 BD2 is denoted above the plots, and residues that were perturbed greater than the average plus two standard deviations after trimming off the top 10% are labelled. A grey line marks this level of significance for each titration.

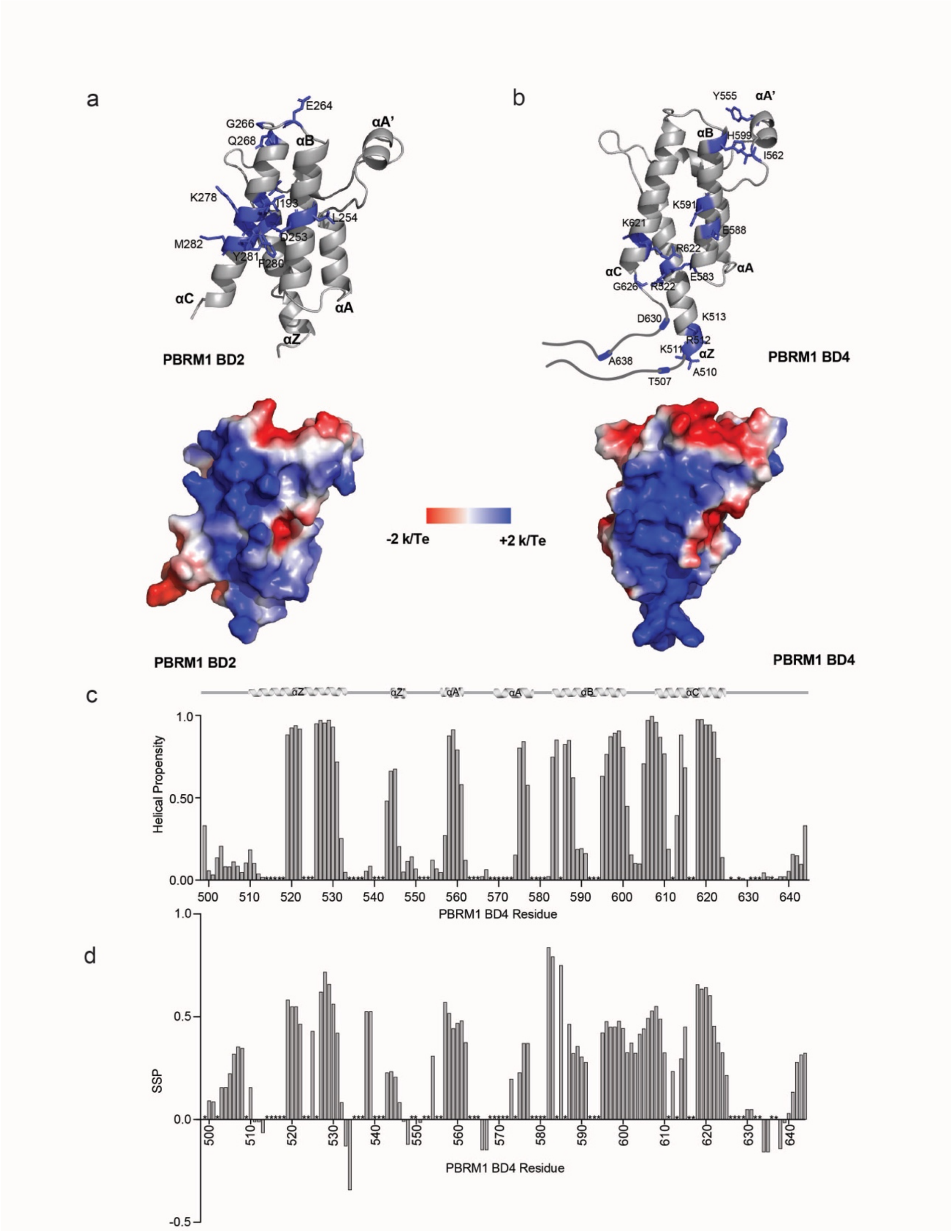


**SI figure 7.** Residues perturbed by addition of dsRNAI for (a) BD2 and (b) BD4 are highlighted as sticks and colored blue on the previously solved structures of each BD (PDB ID 3HMF and 3TLP). Below are the corresponding surface electrostatics calculated in pymol using the APBS plugin. (c) TALOS+ plot and (d) Secondary Structure Prediction (SSP) plot for BD4.

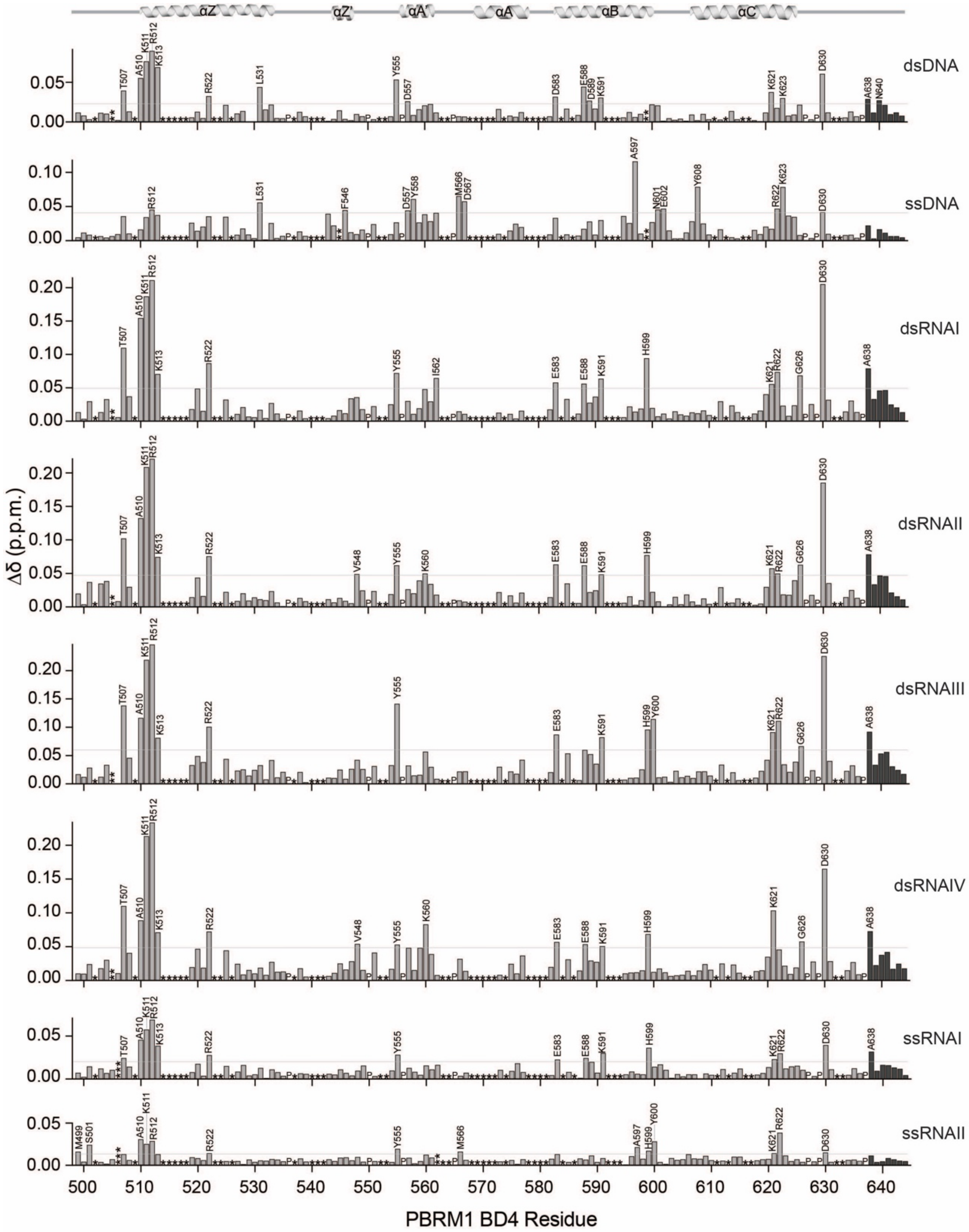


**SI figure 8.** Normalized chemical shift changes between apo and bound (Δδ) for BD4 are plotted as a function of residue upon titration of dsDNAI, ssDNA, dsRNAI, dsRNAII, dsRNAIII, dsRNAIV, ssRNAI and ssRNAII (from top to bottom). Final molar ratios are 1:13.5, 1:24, 1:10, 1:10, 1:10, 1:10, 1:8 and 1:5, respectively. Residues that are unassigned, untrackable due to overlap, or broaden beyond detection upon addition of ligand are marked as (*), (**) and (***) respectively. The secondary structure of the PBRM1 BD4 is denoted above the plots, and residues that were perturbed greater than the average plus two standard deviations after trimming off the top 10% of CSPs are labelled. A grey line marks this level of significance for each titration.

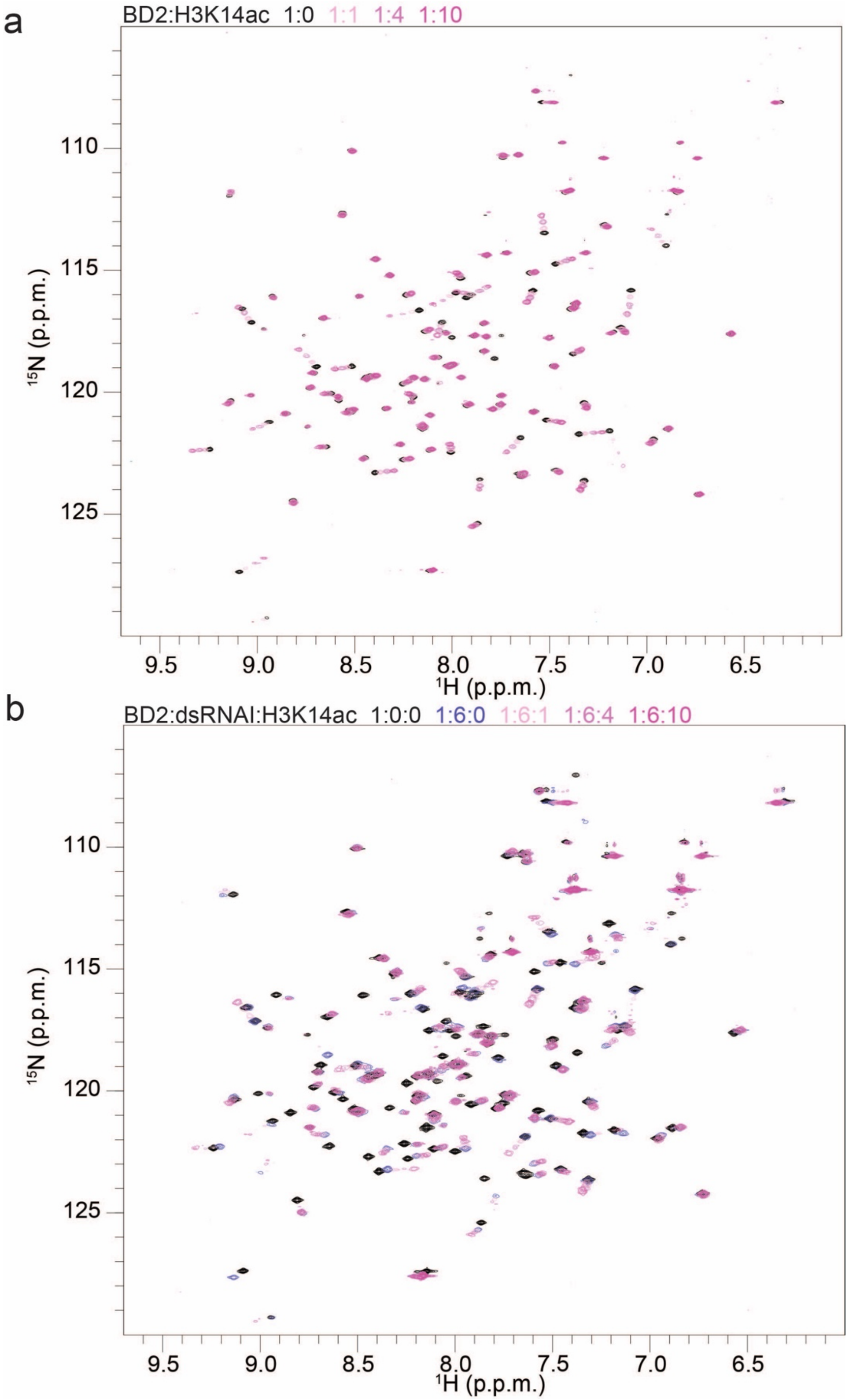


**SI figure 9.** Overlay of $^{1}$H-$^{15}$N HSQC spectra of $^{15}$N-BD2 upon titration of (a) H3K14ac and (b) both dsRNAI and H3K14ac. Spectra are color coded according to legends.

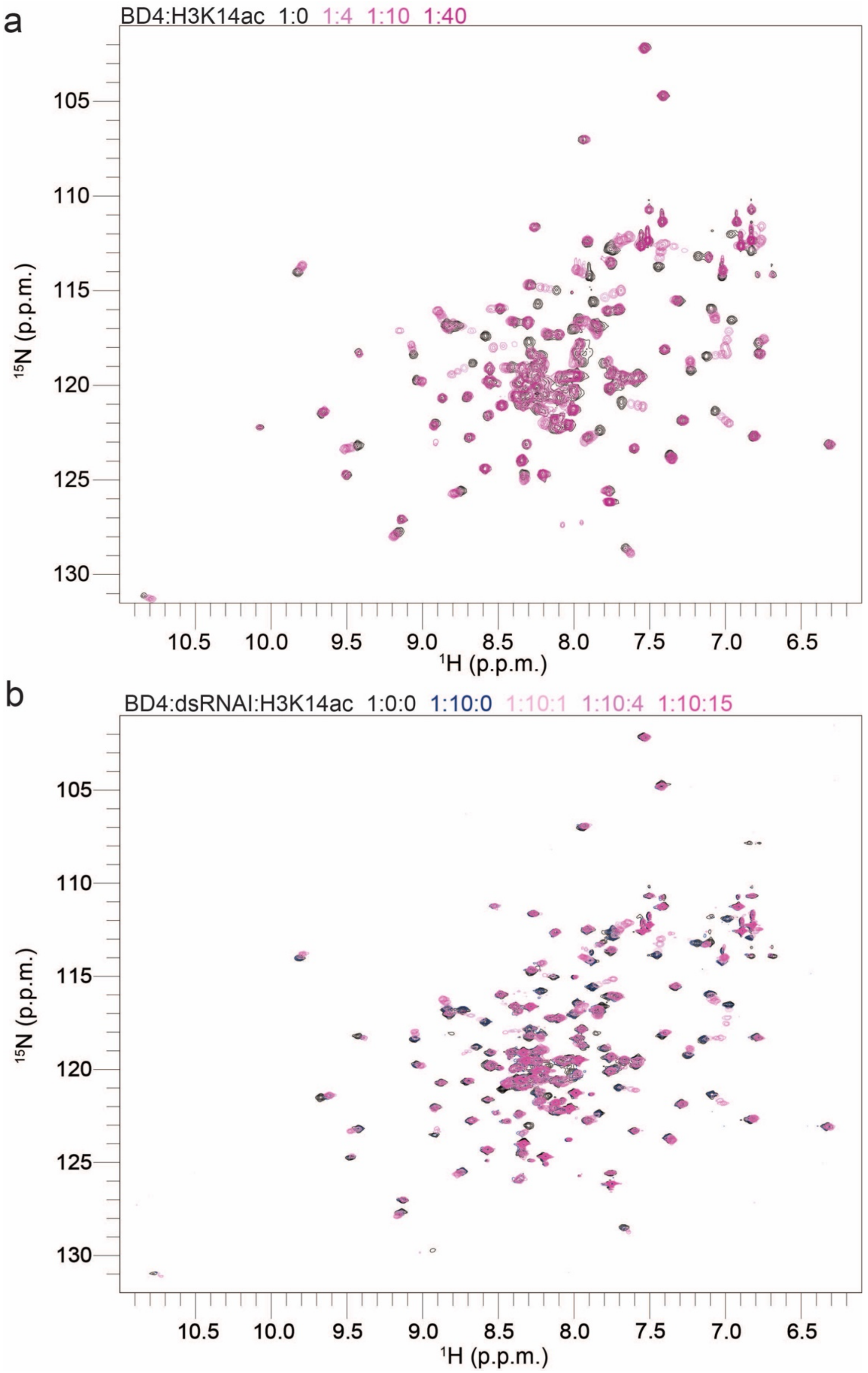


**SI figure 10.** Overlay of $^{1}$H-$^{15}$N HSQC spectra of $^{15}$N-BD4 upon titration of (a) H3K14ac and (b) both dsRNAI and H3K14ac. Spectra are color coded according to legends.

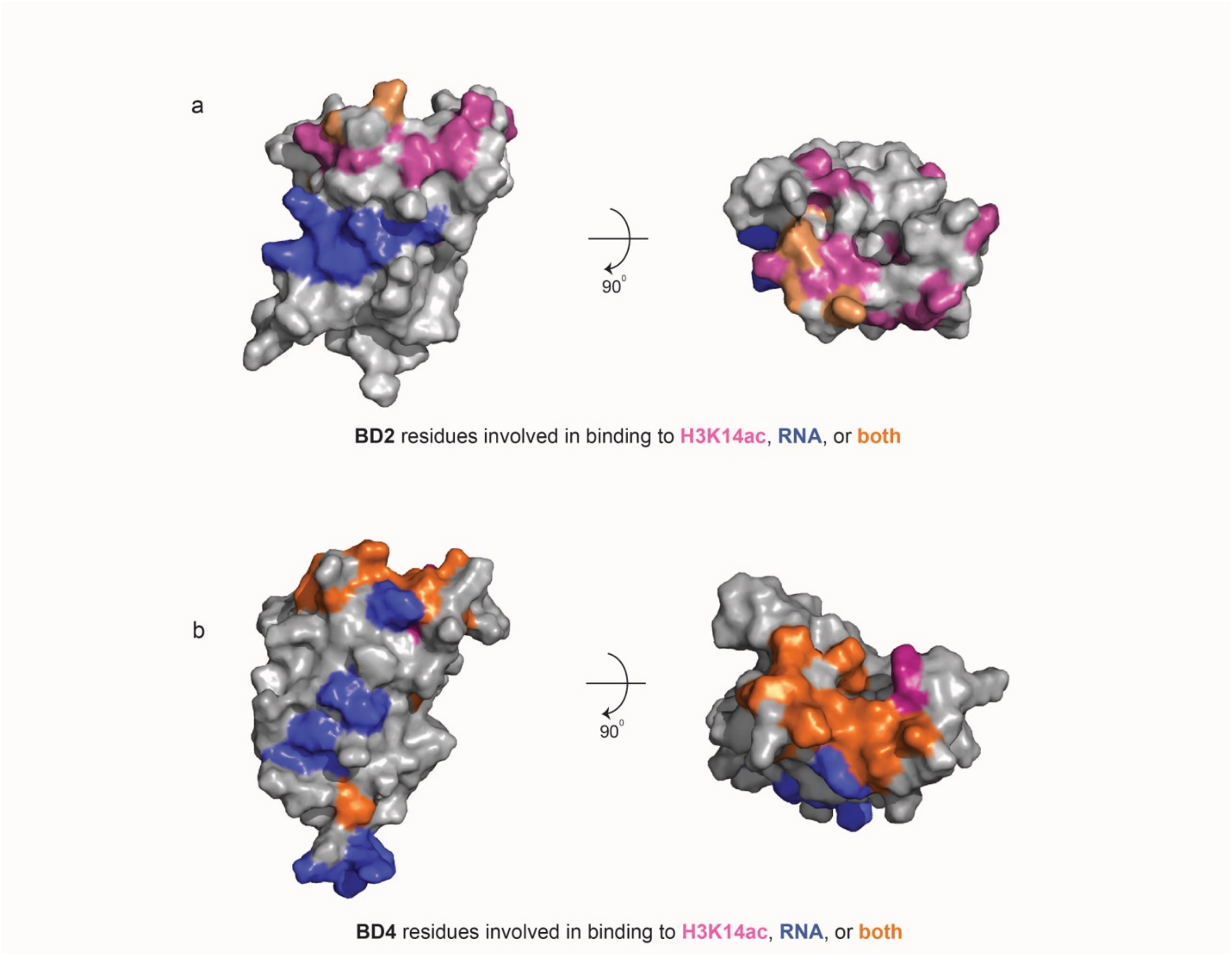


**SI figure 11.** Overlap in the residues that are perturbed upon binding H3K14ac and dsRNAI for BD2 (a) or BD4 (b). A surface representation of BD2 (PDB ID 3HMF) or BD4 (PDB ID 3TLF) with residues colored according to their involvement in binding to H3K14ac peptide (pink), dsRNAI (purple) or both (orange).

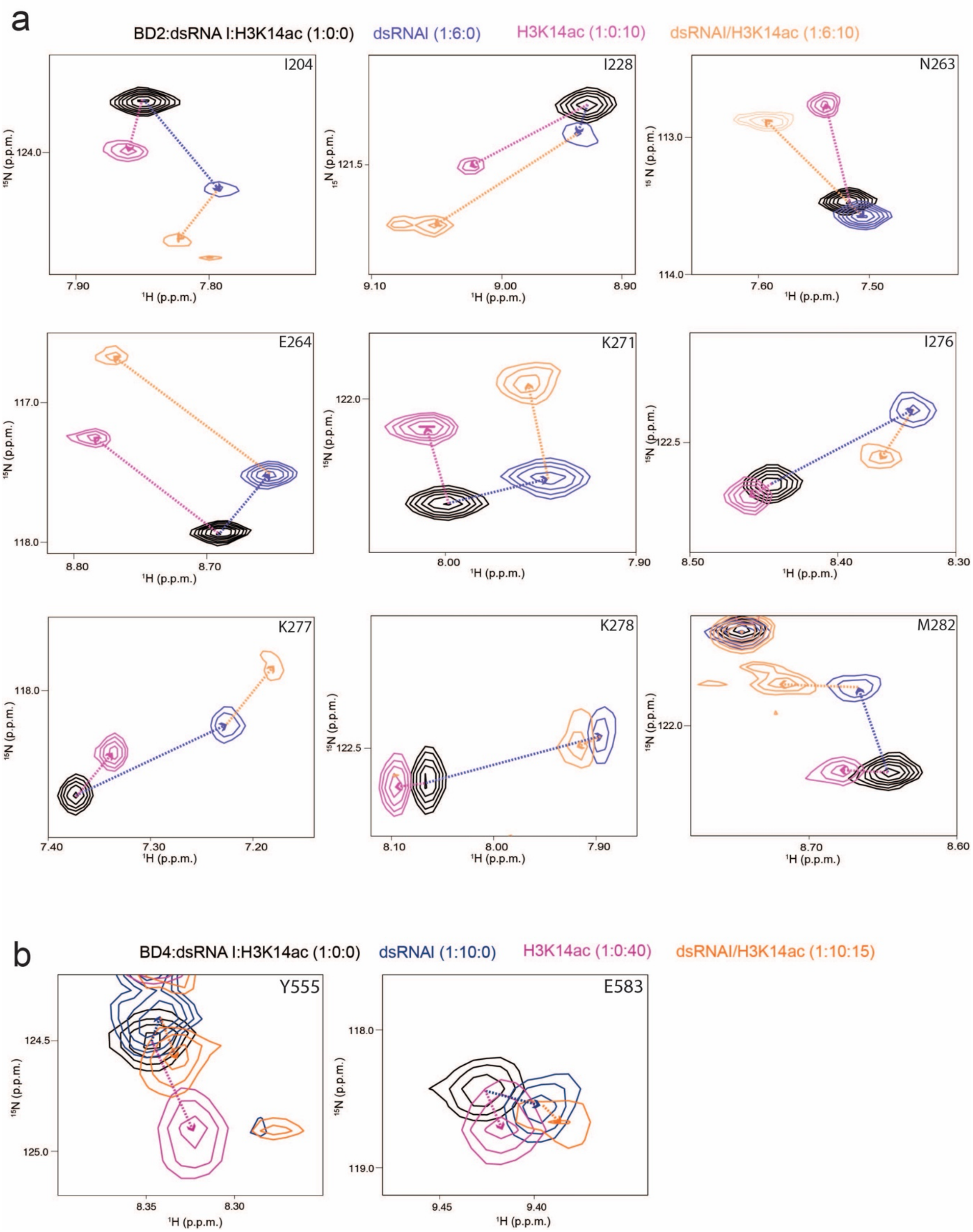


**SI figure 12**. Chemical shift trajectories for selected residues from $^{1}H$-$^{15}N$ HSQC spectral overlays for $^{15}N$-BD2 (a) or $^{15}N$-BD4 (b). Shown are apo (black) in the presence of H3K14ac (pink), dsRNAI (purple), or both dsRNAI and H3K14ac (orange).

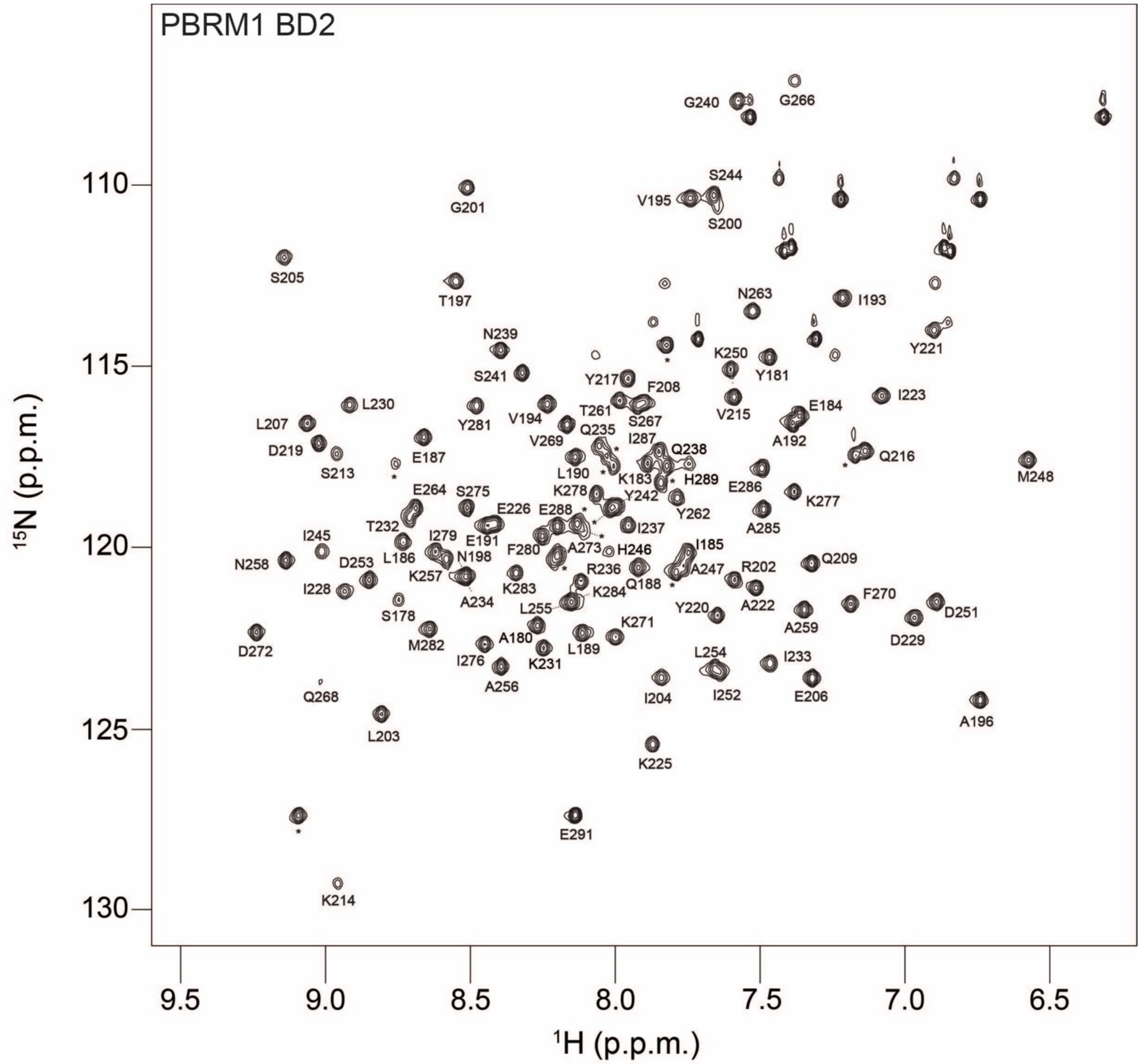


**SI figure 13.** Backbone assignments for BD2. The construct contains 116 residues, 6 of which are prolines. Thus, assuming fast exchange, 110 resonances would be expected. Of the 104 peaks observed 94 were successfully assigned.

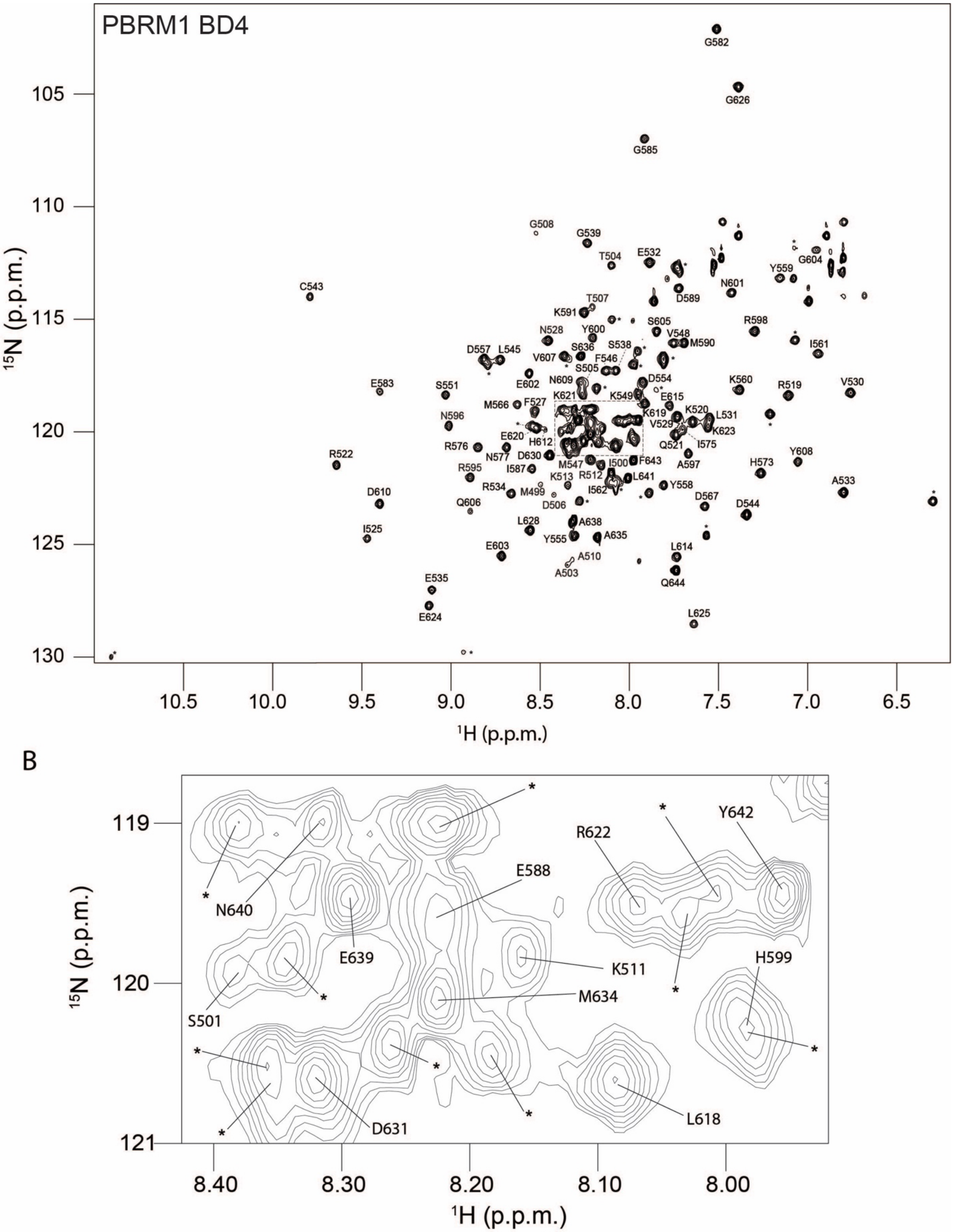


**SI figure 14.** Backbone assignments for BD4. The construct contains 150 residues, 7 of which are prolines. Thus, assuming fast exchange, 143 resonances would be expected. Of the 119 peaks observed 96 were successfully assigned.

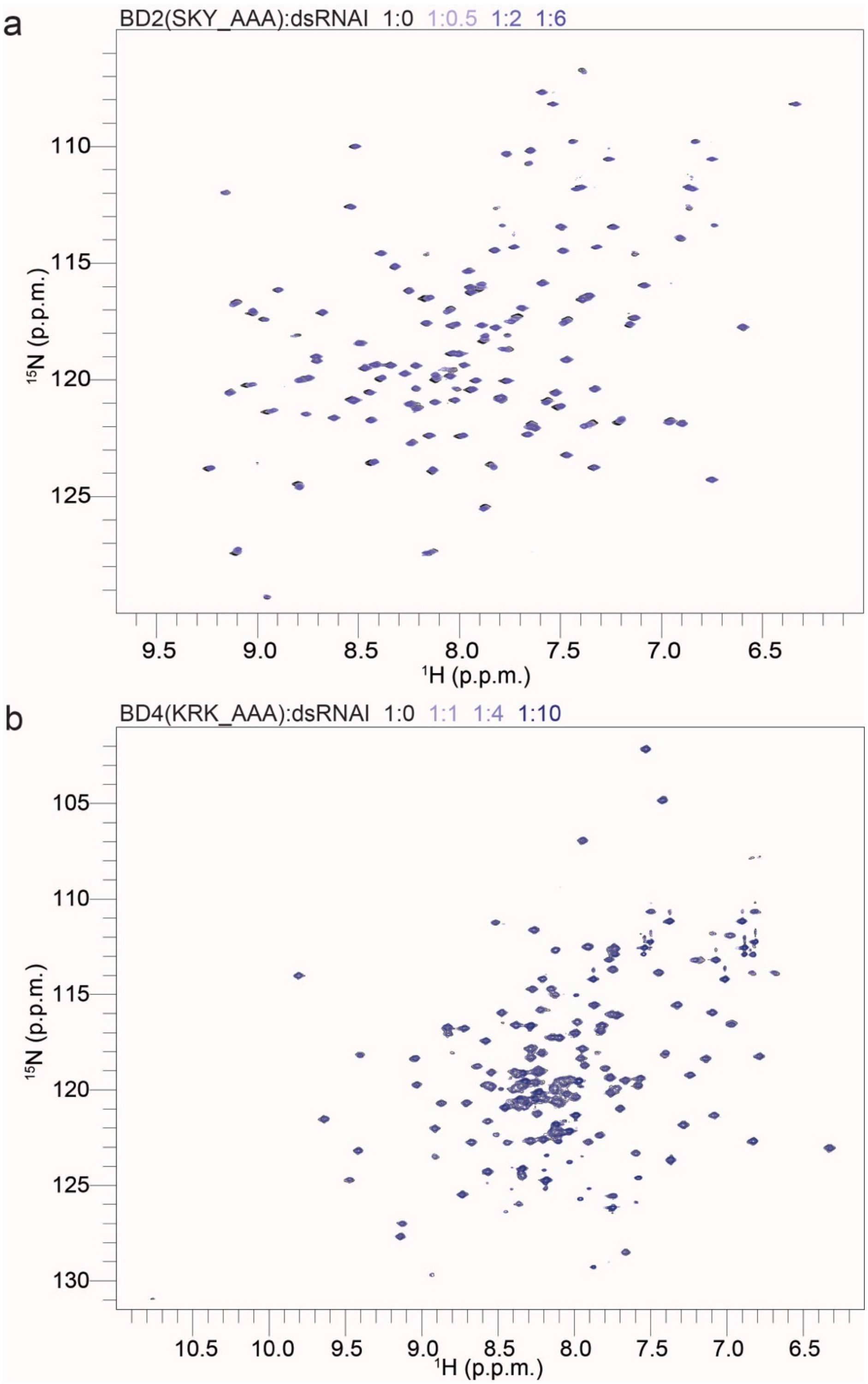


**SI figure 15.** Overlay of $^{1}$H-$^{15}$N HSQC spectra of (a) $^{15}$N-BD2 SKY_AAA or (b) $^{15}$N-BD4 KRK_AAA upon titration of dsRNAI. Spectra are color coded according to protein:dsRNAI molar ratio as shown in the legend. For BD2 spectra were collected at 1:0, 1:0.1, 1:0.25, 1:0.5, 1:1, 1:2, 1:4, 1:6. Protein concentration was 0.1 mM. For BD2 spectra were collected at 1:0, 1:0.5, 1:1, 1:2, 1:3, 1:4, 1:7, 1:10. Protein concentration was 0.1 mM. For clarity, only 4 points are displayed.

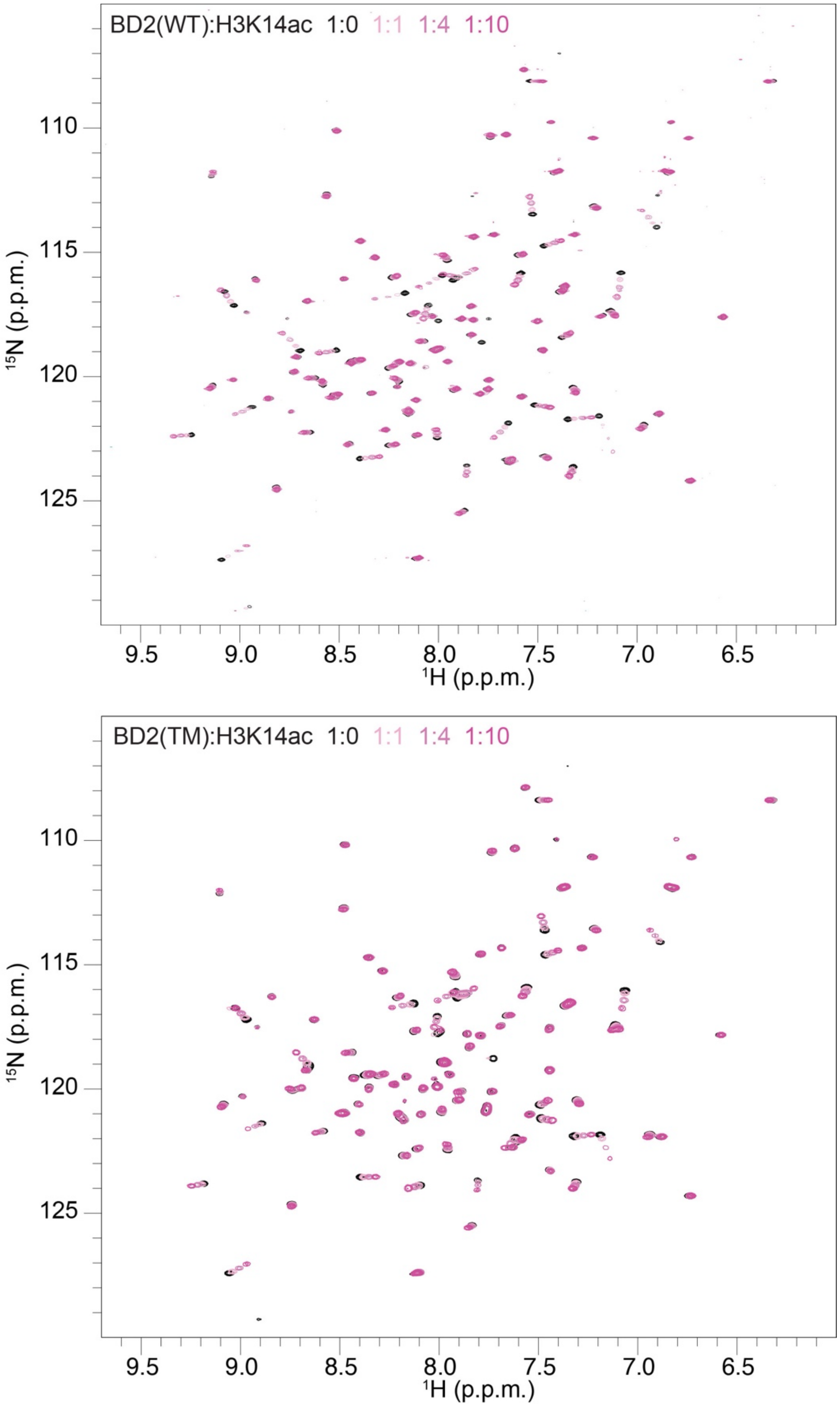


**SI figure 16** Overlay of $^{1}H$-$^{15}N$ HSQC spectra of $^{15}N$-BD2 wild-type (top) or triple mutant (bottom) upon titration of H3K14ac. Spectra are color coded according to legends.

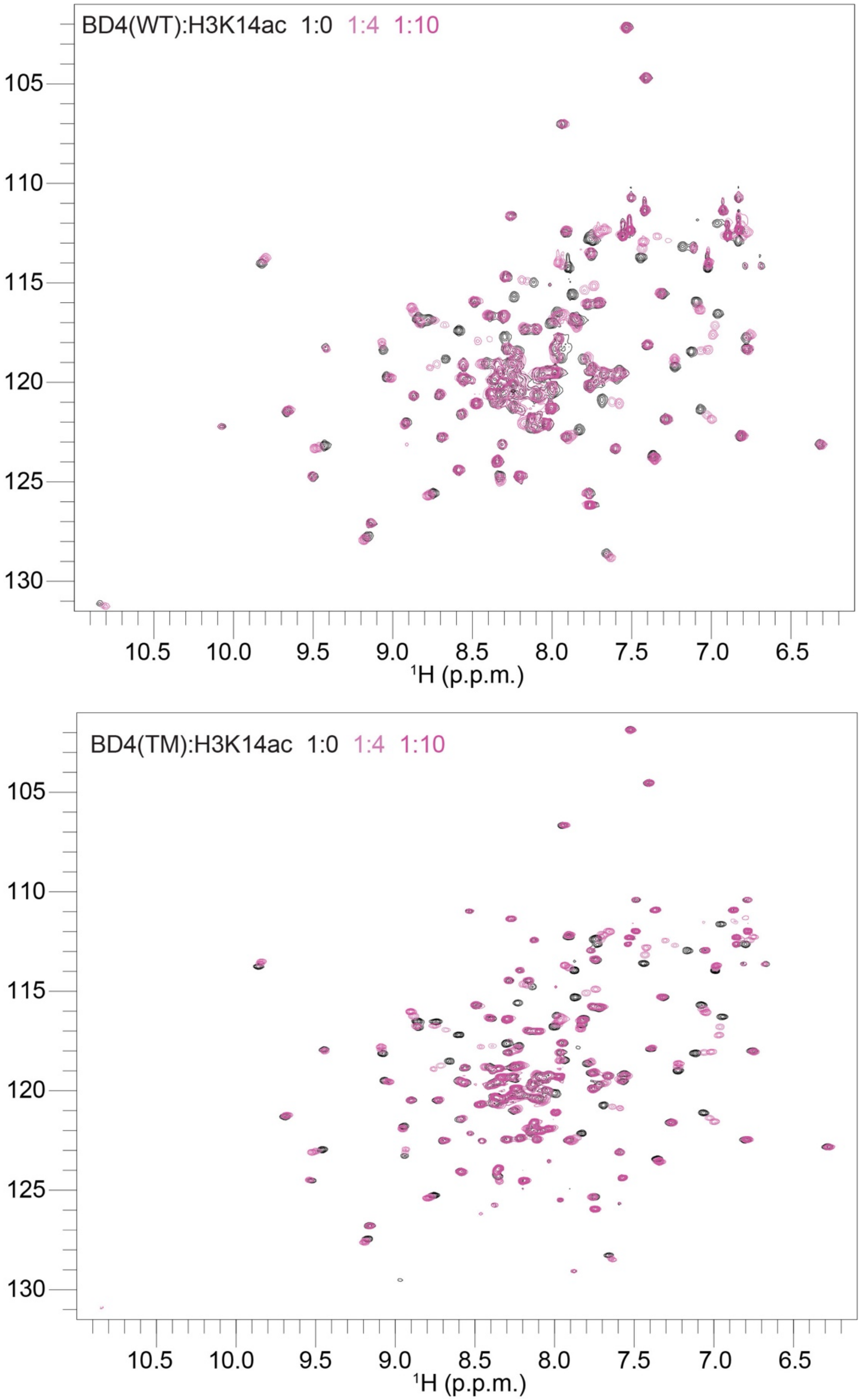


**SI Figure 17** Overlay of $^{1}$H-$^{15}$N HSQC spectra of $^{15}$N-BD4 wild-type (top) or triple mutant (bottom) upon titration of H3K14ac. Spectra are color coded according to legends.

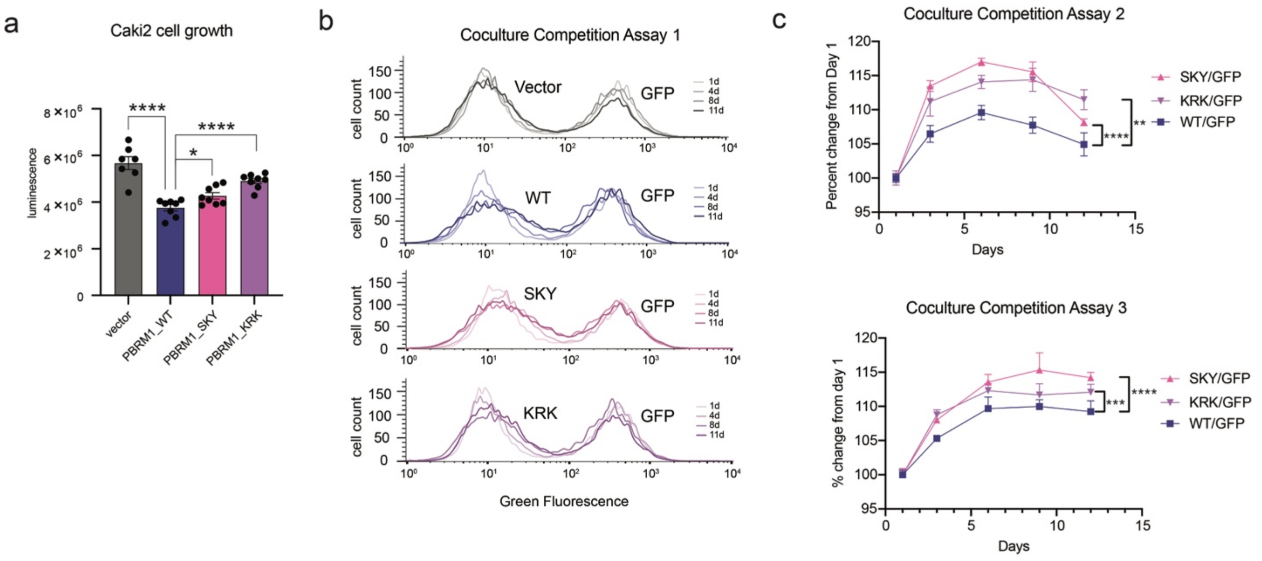


**SI Figure 18.** (a) CellTiter-Glo® measurement of viable cells for seven days of culture of 2,000 cells plated in 96-well plates. A designation of * = p <0.05, ** = p <0.01, *** = p<0.001, **** = p<0.0001 (paired Student *t*-test). Error bars represent s.d. n = 8. (b) Representative histograms of GFP+Caki2 cells and GFP-Caki2 cells with the designated PBRM1 re-expression status. The same initial cell populations were assayed over 11d of culture. (c) The change in the proportion of GFP negative cells compared to GFP positive cells as measured by flow cytometry. Equal numbers of GFP-labeled Caki2 cells and Caki2 cells expressing inducible WT or mutant PBRM1 were plated on day 0 and cells were harvested on designated time points for analysis. A designation of **** = p<0.0001 (paired Student *t*-test). Error bars represent s.d. n = 4.

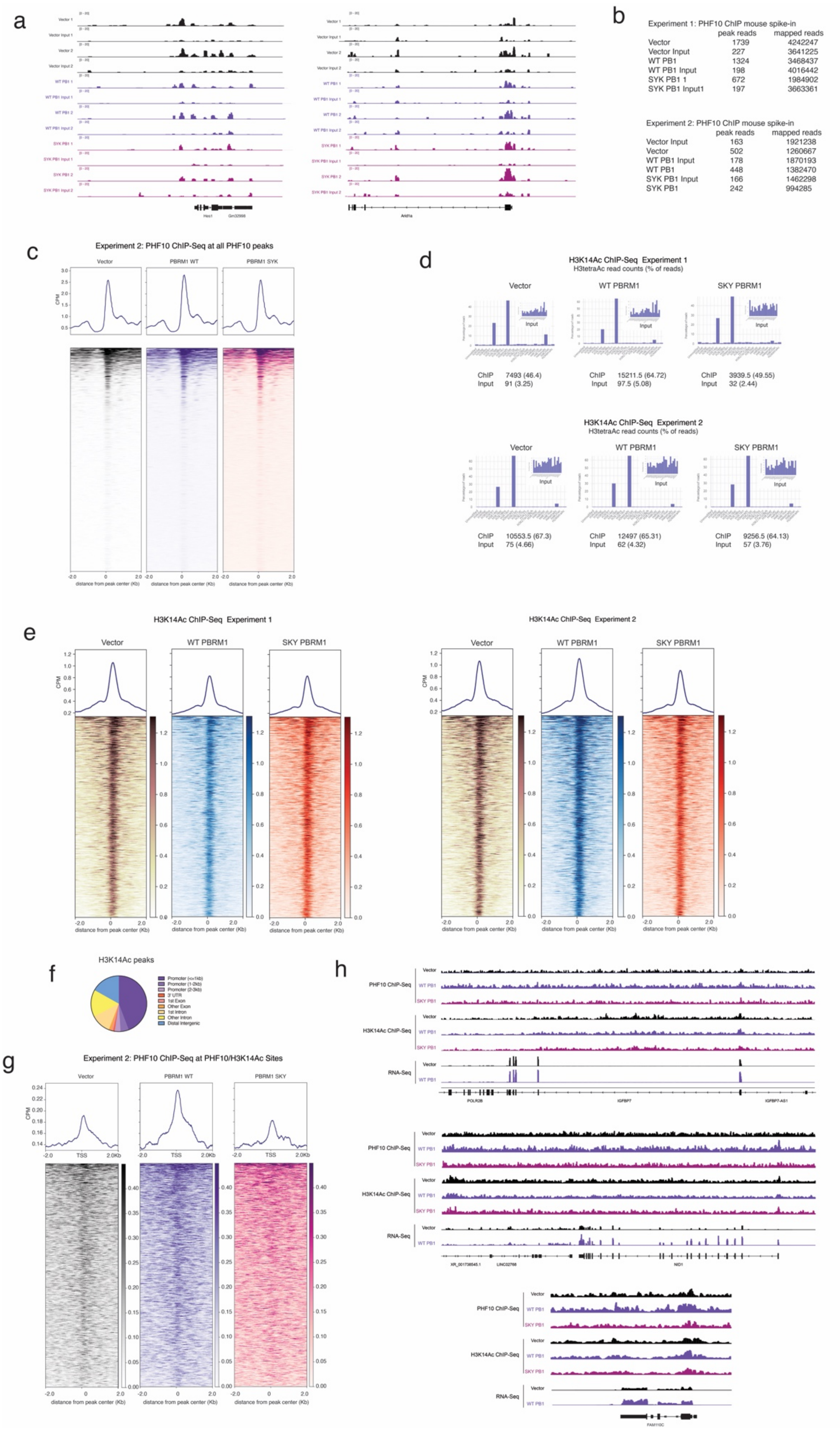

a
Vector 1
Vector Input 1
Vector 2
Vector Input 2
WT PB1 1
WT PB1 Input 1
WT PB1 2
WT PB1 Input 2
SYK PB1 1
SYK PB1 Input 1
SYK PB1 2
SYK PB1 Input 2
Hes1
Gm32688
Arid1a
b
Experiment 1: PHF10 ChIP mouse spike-in
peak reads mapped reads
Vector 1739 4242247
Vector Input 227 3641225
WT PB1 1324 3468437
WT PB1 Input 198 4016442
SYK PB1 1 672 1984902
SYK PB1 Input1 197 3663361
Experiment 2: PHF10 ChIP mouse spike-in
peak reads mapped reads
Vector Input 163 1921238
Vector 502 1260667
WT PB1 Input 178 1870193
WT PB1 448 1382470
SYK PB1 Input 166 1462298
SYK PB1 242 994285
c
Experiment 2: PHF10 ChIP-Seq at all PHF10 peaks
Vector
PBRM1 WT
PBRM1 SYK
CPM
distance from peak center (Kb)
d
H3K14Ac ChIP-Seq Experiment 1
H3tetraAc read counts (% of reads)
Vector
WT PBRM1
SKY PBRM1
Input
ChIP 7493 (46.4)
Input 91 (3.25)
ChIP 15211.5 (64.72)
Input 97.5 (5.08)
ChIP 3939.5 (49.55)
Input 32 (2.44)
H3K14Ac ChIP-Seq Experiment 2
H3tetraAc read counts (% of reads)
Vector
WT PBRM1
SKY PBRM1
ChIP 10553.5 (67.3)
Input 75 (4.66)
ChIP 12497 (65.31)
Input 62 (4.32)
ChIP 9256.5 (64.13)
Input 57 (3.76)
e
H3K14Ac ChIP-Seq Experiment 1
H3K14Ac ChIP-Seq Experiment 2
Vector
WT PBRM1
SKY PBRM1
CPM
distance from peak center (Kb)
f
H3K14Ac peaks
Promoter (<=1kb)
Promoter (1-2kb)
Promoter (2-3kb)
3' UTR
1st Exon
Other Exon
1st Intron
Other Intron
Distal Intergenic
g
Experiment 2: PHF10 ChIP-Seq at PHF10/H3K14Ac Sites
Vector
PBRM1 WT
PBRM1 SKY
CPM
TSS
distance from peak center (Kb)
h
PHF10 ChIP-Seq
H3K14Ac ChIP-Seq
RNA-Seq
Vector
WT PB1
SKY PB1
POLR2B
IGFBP7
IGFBP7-AS1
XR_001738045.1
LINC02768
NID1
FAM110C

**SI Figure 19.** (a) IGV tracks for PHF10 ChIP-Seq sites in spike-in chromatin from mouse NMuMG cells. (b) the number of reads for 30 high confidence PHF10 peaks in the mouse spike-in chromatin in input and ChIP samples. (c) Heat map and metagene analysis at the combined PHF10 peaks from all Caki2 cells. Enrichment at these peaks is calculated for a second PHF10 ChIP-Seq performed in Caki2 cells expressing vector control, WT PBRM1 or PBRM1 with BD2 SKY mutations. (d) Sequencing data from spike-in recombinant acetylated nucleosomes. Sequencing from ChIP (as percentage of spike-in DNA) is represented in large bar graphs and sequencing from input is represented in the inset. Sequencing reads for tetraacetylated nucleosomes are presented below. (e) Heat map and metagene analysis at the overlapping H3K14Ac peaks from all Caki2 cells. Enrichment at these peaks is calculated for two H3K14Ac ChIP-Seq experiments in Caki2 cells expressing vector control, WT PBRM1 or PBRM1 with BD2 SKY mutations. (f) Peak annotation for the overlapping H3K14Ac ChIP-Seq peaks from all Caki2 cell lines. (g) Heat map and metagene analysis at overlapping peaks from H3K14Ac and PHF10 ChIP-Seq, as determined in Figure 6c. Enrichment at these peaks is calculated for a second PHF10 ChIP-Seq performed in Caki2 cells expressing vector control, WT PBRM1 or PBRM1 with BD2 SKY mutations. (h) IGV tracks for ChIP-Seq and RNA-Seq enrichment at IGFBP7, NID1, and FAM110C gene loci in Caki2 cells. RNA-seq data is from (64).

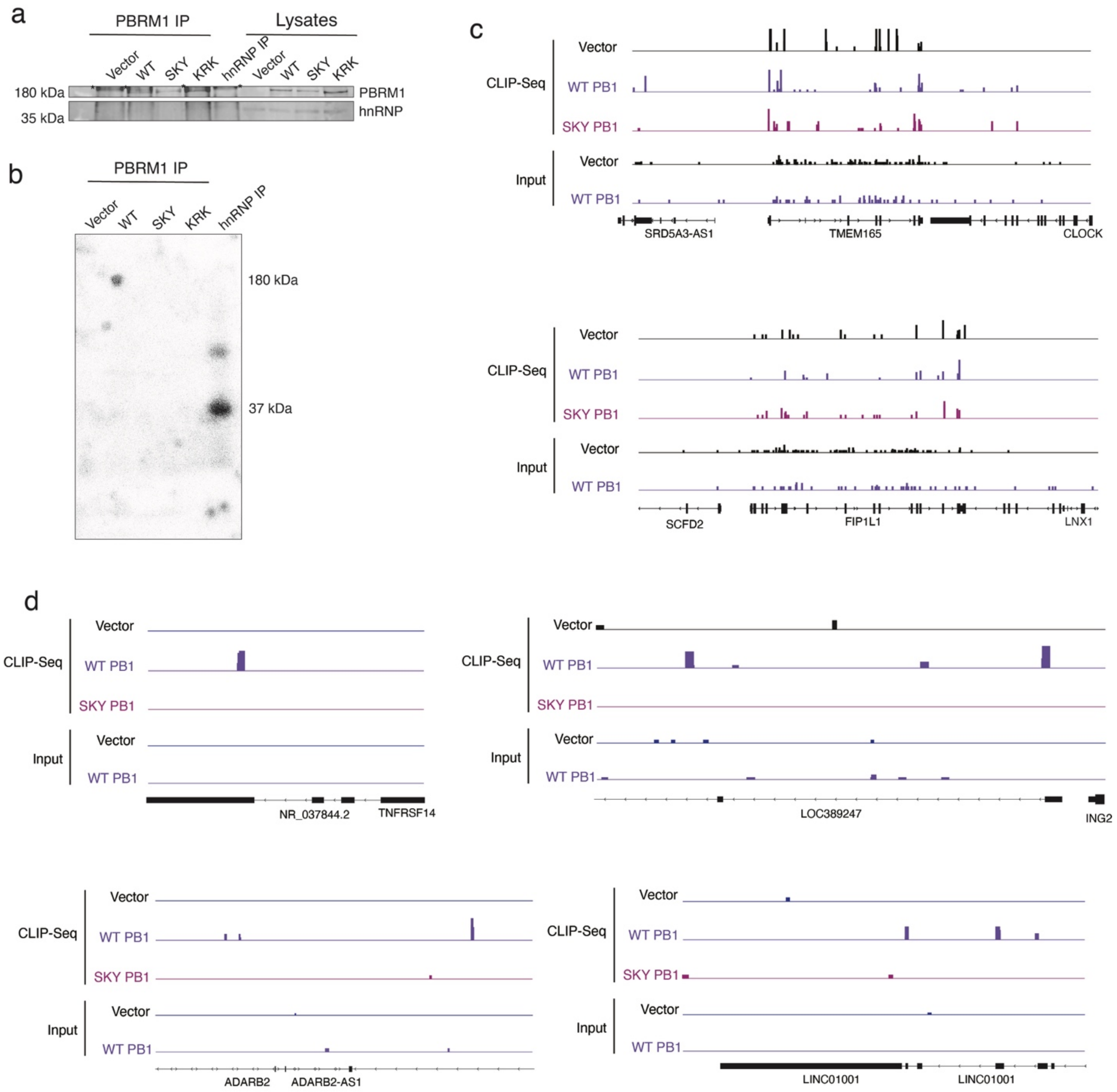


**SI Figure 20. (**a) Immunoblot of immunoprecipitation from UV-crosslinked Caki2 cells (CLIP). * indicates a non-specific band. (b) Phosphorimage of $^{32}$P-labeled immunoprecipitations of exogenous V5 tagged PBRM1, as well as positive control hnRNPA1 from UV-crosslinked Caki2 cells. (c) IGV tracks examples of nonspecific CLIP-Seq enrichment in Caki2 cells. (d) IGV tracks for CLIP-Seq enrichment at ncRNA loci in Caki2 cells.
